# β-Blockers and All-Cause Mortality in Adults with Episodes of Acute Bronchitis: An Observational Study

**DOI:** 10.1371/journal.pone.0067122

**Published:** 2013-06-19

**Authors:** Frans H. Rutten, Rolf H. H. Groenwold, Alfred P. E. Sachs, Diederick E. Grobbee, Arno W. Hoes

**Affiliations:** Department Julius Center for Health Sciences and Primary Care, University Medical Center Utrecht, Utrecht, The Netherlands; Cardiff University, United Kingdom

## Abstract

**Background:**

Recent observational studies suggest that β-blockers may improve long-term prognosis in patients with chronic obstructive pulmonary disease (COPD). We assessed whether β-blocker use improves all-cause mortality in patients with episodes of acute bronchitis.

**Methods:**

An observational cohort study using data from the electronic medical records of 23 general practices in the Netherlands. The data included standardized information about daily patient contacts, diagnoses, and drug prescriptions. Cox regression was applied with time-varying treatment and covariates.

**Results:**

The study included 4,493 patients aged 45 years and older, with at least one episode of acute bronchitis between 1996 and 2006. The mean (SD) age of the patients was 66.9 (11.7) years, and 41.9% were male. During a mean (SD) follow up period of 7.7 (2.5) years, 20.4% developed COPD. In total, 22.7% had cardiovascular comorbidities, resulting in significant higher mortality rates than those without (51.7% vs. 12.0%, p<0.001). The adjusted hazard ratio of cardioselective β-blocker use for mortality was 0.62 (95% confidence interval [CI], 0.50–0.77), and 1.01 (95% CI 0.75–1.36) for non-selective ones. Some other cardiovascular drugs also reduced the risk of mortality, with adjusted HRs of 0.60 (95% CI 0.46–0.79) for calcium channel blockers, 0.88 (95% CI 0.73–1.06) for ACE inhibitors/angiotensin receptor blockers, and 0.42 (95% CI 0.31–0.57) for statins, respectively.

**Conclusion:**

Cardiovascular comorbidities are common and increase the risk of mortality in adults with episodes of acute bronchitis. Cardioselective β-blockers, but also calcium channel blockers and statins may reduce mortality, possibly as a result of cardiovascular protective properties.

## Introduction

Acute bronchitis is a very common pulmonary illness, affecting 44 out of 1,000 adults older than 16 years annually, with 82 percent of episodes occurring in fall or winter.[1] Acute bronchitis is a typical clinical diagnosis, lasting 1 to 3 weeks, and diagnosed on the basis of cough, occasionally dyspnea, sputum, and wheeze in combination with rhonchi or coarse rales on pulmonary auscultation.[1]–[3] Treatment with antibiotics is still the mainstream [4], although, meta-analyses of randomized, controlled trials conclude that routine antibiotic treatment does not provide major clinical benefit [5]–[7]. Respiratory viruses are also suspected, although ‘no isolated pathogen’ is a frequent finding [8]–[10]. Moreover, bronchial hyper-responsiveness seems to play a crucial role, being present in one-third to over 50% of patients [8], [11]–[13]. A prospective study showed that one-third of adults with episodes of acute bronchitis eventually developed asthma or chronic obstructive pulmonary disease (COPD) [3]. The perspective that having episodes of acute bronchitis implicates a more chronic disease, and that affected adults could at least partly be considered as ‘pre-COPD patients’ has not received much attention in literature. In line with this, (cardiovascular) comorbidities have not been considered as treatment targets, nor has all-cause mortality been considered as an important outcome. Time has come to do so, because multiple recent observational studies suggested that cardiovascular drugs, especially ß-blockers and statins may reduce all-cause mortality in patients with COPD [14]–[18]. Whether cardiovascular drugs may improve survival in adults with episodes of acute bronchitis has never been studied.

We therefore wanted to assess whether the use of ß-blockers and similar cardiovascular drugs may improve long-term survival in adults with at least one episode of acute bronchitis.

## Methods

### Study population

To study the effects of β-blocker therapy and some other cardiovascular drugs on the risk for all-cause mortality in adult patient with at least one episode of acute bronchitis we used data from the computerised medical database of the General Practitioner Research Network (HNU) of the University Medical Center Utrecht, the Netherlands. This database includes cumulative information on a dynamic cohort of approximately 60,000 patients enlisted with 33 general practitioners. All patient contacts with the general practitioner are recorded in the electronic medical file using the International Classification of Primary Care (ICPC-2) coding system, and prescriptions are coded according to the Anatomical Therapeutical Chemical Classification (ATC) coding system [19], [20]. All primary care out of office hours patient contacts and specialist letters with information about hospital admissions and findings from outpatient clinics are also copied in the database and labelled with an ICPC-2 code. All citizens are registered with a general practitioner in the Netherlands, irrespective of treatment by a medical specialist, except for those living in a nursing home. Medical specialists in the Netherlands routinely provide information (usually by letter) to the general practitioners about contacts with the GP's their patients, including when applicable, notification of death. Date of death and its suspected cause are always notified in the GP patient file.

For the present study, all patients aged 45 years and older that had experienced at least one episode of acute bronchitis (ICPC-2 code R78) between January 1, 1995 and December 31, 2005 were included, starting from the moment of the first episode of acute bronchitis. Eligible patients were followed up until they died (study end point) or moved or until the end of the study period (December 31, 2005), whichever came first. Those who moved during the study period were censored and contributed no person time or events beyond that time. Acute bronchitis (ICPC-2 code R78) as a clinical diagnosis was established by a physician when patients had (subacute) coughing in combination with rhonchi or coarse rales on pulmonary auscultation, with or without fever, and when other pulmonary diagnoses, e.g. (exacerbation of) asthma or COPD were not considered applicable [20].

The study was conducted in accordance with the Law for the Protection of Personal Data and confirmed to the Declaration of Helsinki. The Medical Ethical Committee of the University Medical Center Utrecht approved our study and waived the need for written informed consent from participants because only de-identified patient data were used in the study.

### Assessment of exposure, outcome, and confounders

Assessment of β-blocker exposure was based on ATC coding (ATC code C07) [19]. A distinction was made between cardio-selective and non-selective β-blockers. Vital status was based on the medical records in the GP registration. We considered the following variables as potential confounders: age, sex, history of cardiovascular disease (i.e. angina pectoris, myocardial infarction, coronary artery bypass grafting, percutaneous coronary intervention, atrial fibrillation, heart failure, peripheral arterial disease, or stroke), hypertension, diabetes mellitus, cardiovascular drug use others than β-blockers, and pulmonary drug use. Other cardiovascular drugs, with similar indications as β-blockers, i.e., ischemic heart disease and hypertension were also considered, and included calcium channel blockers, angiotensin converting enzyme (ACE) inhibitors or angiotensin receptor blockers (ARBs), and statins. Every time we evaluated a cardiovascular drug, we adjusted for cardiovascular drug use others than the one under investigation. Cardiovascular drug prescriptions prescribed before and after the first episode of acute bronchitis were considered in our analyses.

### Data analysis

Cox proportional hazards regression analyses were used to calculate crude (unadjusted) and adjusted hazard ratios (HRs) and their 95% confidence intervals (CI) of the risk of all-cause mortality with the use of β-blockers. To account for immortal time bias, β-blocker use was included as a time-varying variable in the Cox model. Adjustment for the aforementioned potential confounders was done as time-depending covariates in the Cox model [21].

To address the possibility of (residual) confounding by indication, we performed subgroup analyses, including patients (1) known with a cardiovascular disease, or (2) who developed COPD, that is, who got a diagnosis of COPD *after* the first episode of acute bronchitis.

Similar analyses were performed with statins, ACE-inhibitors or angiotensin receptor blockers, and calcium-channel blockers.

All analyses were conducted in SPSS for Windows version 17.0 (SPSS Inc., Chicago, Illinois, USA), and R for Windows version 2.15.0.

## Results

A total of 4,493 patients 45 years and older with one or more episodes of acute bronchitis were included in the study. The mean (SD) age of the patients at the start of the study was 66.9 (11.7) years, and 41.9% were male. During the mean (SD) follow-up period of 7.7 (2.5) years, 943 patients (21.0%) died: 10.2% of those who ever used a ß-blocker compared to 26.2% of those who never used a ß-blocker (p<0.001). Patient characteristics according to ß-blocker use are presented in Table 1.

**Table 1:** Characteristics of 4,493 patients 45 years or older with a diagnosis of acute bronchitis according to β-blocker use.

*Characteristics*	Ever β-blocker use (N = 1,460)	Never β-blocker use (N = 3,033)	p-value**
Mean age (SD) in years*	67.7 (10.9)	66.5 (12.2)	<0.001
Male sex	586 (40.1)	1,298 (42.8)	0.09
COPD	272 (18.6)	646 (21.3)	0.04
***Cardiovascular risk factors***			
Hypertension	1,009 (69.1)	692 (22.8)	<0.001
Diabetes	323 (22.1)	400 (13.2)	<0.001
***Cardiovascular diseases***			
Angina pectoris	368 (25.2)	227 (7.5)	<0.001
Prior myocardial infarction	103 (7.1)	52 (1.7)	<0.001
Atrial fibrillation	252 (17.3)	125 (4.1)	<0.001
Heart failure	313 (21.4)	395 (13.0)	<0.001
Stroke	112 (7.7)	158 (5.2)	0.002
Peripheral arterial disease	124 (8.5)	133 (4.4)	<0.001
***Cardiovascular drug use***			
β-blockers	NA	NA	NA
Cardioselective	1,170 (80.1)	NA	NA
Non-selective	290 (19.9)	NA	NA
ACE inhibitors or ARBs	733 (50.2)	597 (19.7)	<0.001
Aldosterone antagonists §	134 (9.2)	124 (4.1)	<0.001
Statins	483 (33.1)	370 (12.2)	<0.001
Diuretics	938 (64.2)	965 (31.8)	<0.001
Nitrates	441 (30.2)	279 (9.2)	<0.001
Calcium channel blockers	463 (31.7)	294 (9.7)	<0.001
Digoxin	162 (11.1)	191 (6.3)	<0.001
Acetyl salicylate or clopidrogel	362 (24.8)	282 (9.3)	<0.001
Vitamin K antagonists	390 (26.7)	337 (11.1)	<0.001
***Pulmonary drug use***			
Beta2-mimetics inhalers	407 (27.9)	1,049 (34.6)	<0.001
Anticholinergic inhalers	507 (34.7)	928 (30.6)	0.006
Inhalation corticosteroids	458 (31.4)	1,010 (33.3)	0.215

Values are in numbers and percentages unless stated otherwise.

Abbreviation: NA; not applicable.

* At study entry.

** based on t-test or Fisher exact test as appropriate.

§ Aldosterone antagonists are spironolactone and eplerenone.

In total, 1,020 patients (22.7%) had cardiovascular comorbidities, including ischemic heart disease (angina pectoris, prior myocardial infarction, percutaneous coronary intervention, or coronary artery bypass grafting), heart failure, atrial fibrillation, peripheral arterial disease and stroke. These patients had significant higher mortality rates than those without overt cardiovascular diseases (51.7% vs 12.0%, p<0.001).

Cardiovascular drug prescriptions was high (Table 1), and 1,460 patients (32.5%) were prescribed β-blockers, mainly cardioselective ones (26.0%).

### All-cause mortality

The crude and adjusted HRs based on the multivariable Cox hazard model for β-blocker use were 1.00 (95% CI, 0.84–1.19) and 0.71 (95% CI, 0.58–0.84), respectively. For cardioselective and non-selective β-blockers the adjusted HRs were 0.62 (95% CI, 0.50–0.77) and 1.01 (95% CI, 0.75–1.36), respectively (Table 2).

**Table 2:** Crude and adjusted hazard ratios (HR) for all-cause mortality according to β-blocker use in 4,493 patients aged 45 years or over with a diagnosis of acute bronchitis.

	Hazard ratio (95% Confidence Interval)		
Variable	Any β-blocker	Cardioselective β-blocker	Nonselective β-blocker
Unadjusted (crude)	1.00 (0.84–1.19)	0.89 (0.72–1.09)	1.37 (1.02–1.84)
Covariates included cumulatively in the Cox model to calculate adjusted HRs +			
Age	0.77 (0.65–0.92)	0.69 (0.56–0.85)	1.03 (0.77–1.38)
Sex	0.79 (0.67–0.95)	0.70 (0.57–0.86)	1.11 (0.83–1.49)
diabetes, hypertension, CV diseases	0.68 (0.57–0.81)	0.60 (0.48–0.74)	0.96 (0.71–1.29)
use of CV drugs other than β-blocker	0.68 (0.56–0.81)	0.59 (0.48–0.74)	0.95 (0.71–1.28)
COPD	0.70 (0.58–0.84)	0.61 (0.50–0.76)	1.00 (0.74–1.35)
use of pulmonary drugs	0.71 (0.58–0.84)	0.62 (0.50–0.77)	1.01 (0.75–1.36)

Adjusted HRs based on Cox proportional hazards were calculated step by step after adjustment for age, sex, diabetes, hypertension, cardiovascular diseases, other cardiovascular drugs than the one under study, COPD, use of pulmonary drugs.

Cardiovascular drugs include β-blockers, angiotensin-converting enzyme inhibitors, angiotensin receptor blockers, aldosterone antagonists, statins, digoxin, loop and thiazide diuretics, nitrates, aspirin and clopidrogel, vitamin-K antagonists, and calcium channel blockers.

Pulmonary drugs include inhalers of β2-agonists, anticholinergic agents, corticosteroids, and oral xanthine derivates.

For calcium channel blockers, ACE-inhibitors or angiotensin blockers, and statins, the adjusted HRs were 0.60 (95% CI, 0.46–0.79), 0.88 (95% CI, 0.73–1.06), and 0.42 (95% CI, 0.31–0.57), respectively (Table 3).

**Table 3:** Crude and adjusted hazard ratios (HR) for mortality according to statin use, angiotensin-converting enzyme inhibitor or angiotensin receptor blocker use, and calcium channel antagonists use in 4,493 patients with a diagnosis of acute bronchitis.

	Hazard ratio (95% Confidence Interval)		
Variable	CCB	ACE-I or ARB	Statin
Unadjusted (crude)	0.99 (0.77–1.29)	1.31 (1.11–1.54)	0.49 (0.37–0.66)
Covariates included cumulatively in the Cox model to calculate adjusted HRs +			
Age	0.70 (0.54–0.90)	1.04 (0.88–1.23)	0.57 (0.42–0.76)
Sex	0.69 (0.53–0.90)	1.04 (0.88–1.23)	0.54 (0.40–0.72)
diabetes, hypertension, CV diseases	0.61 (0.47–0.80)	0.90 (0.75–1.07)	0.42 (0.31–0.56)
use of CV drugs other than β-blocker	0.62 (0.47–0.80)	0.91 (0.76–1.09)	0.42 (0.31–0.56)
COPD	0.60 (0.47–0.79)	0.89 (0.74–1.06)	0.42 (0.31–0.57)
use of pulmonary drugs	0.60 (0.46–0.79)	0.88 (0.73–1.06)	0.42 (0.31–0.57)

Abbreviations: CCB; calcium channel blocker, ACE-I; angiotensin-converting enzyme inhibitor, ARB; angiotensin receptor blocker, COPD; chronic obstructive pulmonary disease.

Adjusted HRs based on Cox proportional hazards were calculated step by step after adjustment for age, sex, diabetes, hypertension, cardiovascular diseases, other cardiovascular drugs than the one under study, COPD, use of pulmonary drugs.

Cardiovascular drugs include β-blockers, angiotensin-converting enzyme inhibitors, angiotensin receptor blockers, aldosterone antagonists, statins, digoxin, loop and thiazide diuretics, nitrates, aspirin and clopidrogel, vitamin-K antagonists, and calcium channel blockers.

Pulmonary drugs include inhalers of β2-agonists, anticholinergic agents, corticosteroids, and oral xanthine derivates.

### All-cause mortality in subgroups

In the subgroup of patients with overt cardiovascular disease (n = 1,020), 527 (51.7%) patients died. The adjusted HRs for cardioselective and non-selective β-blockers in this subgroup were similar to those without cardiovascular disease, with values of 0.63 (95% CI 0.48–0.83) and 1.09 (95% CI, 0.74–1.61), respectively (Table 4).

**Table 4:** Time-dependent analysis with adjusted hazard ratios (HR) for all-cause mortality according to β-blocker use in subgroups of patients with a diagnosis of acute bronchitis.

	All ß-blockers			Cardioselective ß-blockers		Nonselective ß-blockers	
	*Treated*	*Untreated*		*Treated*		*Treated*	
*Subgroup*	*Events/Follow-up time (py)*	*Events/Follow-up time (py)*	*HR (95% CI)*	*Events/Follow-up time (py)*	*HR (95% CI)*	*Events/Follow-up time (py)*	*HR (95% CI)*
Patients *without* overt cardiovascular disease†	45/1915	371/14260	0.82 (0.59; 1.14)	31/1462	0.74 (0.50; 1.09)	14/452	1.05 (0.61; 1.81)
Patients *with* overt cardiovascular disease†	104/1810	423/5650	0.73 (0.58; 0.93)	71/1403	0.63 (0.48; 0.83)	33/406	1.09 (0.74; 1.61)
Patients *without* COPD§	97/2857	517/13945	0.64 (0.51; 0.81)	63/2184	0.55 (0.41; 0.72)	34/673	0.94 (0.65; 1.37)
Patients *with* COPD§	52/868	277/5964	0.80 (0.58; 1.11)	39/682	0.73 (0.51; 1.06)	13/185	1.09 (0.62; 1.91)

Abbreviations: HR, hazard ratio; CI, Confidence interval.

† Overt cardiovascular disease was defined as ischemic heart disease, heart failure, peripheral arterial disease, or stroke during follow-up.

§ COPD was defined as a diagnosis of COPD (clinically or based on spirometry) during follow-up.

In the subgroup of patients who developed COPD similar HRs cardioselective and non-selective β-blockers for all-cause mortality were found as in those who did not develop COPD during 7.7 years follow-up, with values of 0.55 (95% CI 0.41–0.72) and 0.94 (95% CI 0.65–1.37), respectively (Table 4).

Subgroup analyses with calcium channel blockers, ACE-inhibitors or ARBs, and statins revealed that the effects on all-cause mortality were similar for each drug in the subgroups as was seen in the whole population. Only calcium channel blockers seemed to be more effective in patients with as compared to those without cardiovascular comorbidity (Table 5).

**Table 5:** Time-dependent analysis with adjusted hazard ratios (HR) for all-cause mortality according to calcium channel blocker, ACE inhibitor or angiotensin receptor blocker, or statin use in subgroups of patients with a diagnosis of acute bronchitis.

	Calcium channel blocker use			ACE inhibitor or angiotensin receptor blocker use			Statin use		
	*Treated*	*Untreated*		*Treated*	*Untreated*		*Treated*	*Untreated*	
*Subgroup*	*Events/Follow-up time (py)*	*Events/Follow-up time (py)*	*HR (95% CI)*	*Events/Follow-up time (py)*	*Events/Follow-up time (py)*	*HR (95% CI)*	*Events/Follow-up time (py)*	*Events/Follow-up time (py)*	*HR (95% CI)*
Patients *without* overt cardiovascular disease†	20/629	396/15,545	0.78 (0.48; 1.28)	48/1711	368/14,464	0.80 (0.56; 1.15)	14/1,184	402/14,990	0.47 (0.28; 0.80)
Patients *with* overt cardiovascular disease†	40/879	487/6,580	0.54 (0.40; 0.75)	125/1748	402/5,711	1.00 (0.79; 1.28)	34/1,116	493/6,344	0.47 (0.32; 0.68)
Patients *without* COPD§	37/954	577/15,847	0.65 (0.47; 0.91)	98/2230	516/14,572	0.88 (0.68; 1.15)	27/1,665	587/15,137	0.36 (0.24; 0.55)
Patients *with* COPD§	23/554	306/6,278	0.54 (0.35; 0.84)	75/1229	254/5,603	0.89 (0.66; 1.20)	21/635	308/6,197	0.49 (0.31; 0.79)

Abbreviations: HR, hazard ratio; CI, Confidence interval.

† Overt cardiovascular disease was defined as ischemic heart disease, heart failure, peripheral arterial disease, or stroke during follow-up.

§ COPD was defined as a diagnosis of COPD (clinically or based on spirometry) during follow-up.

## Discussion

To our knowledge, this is the first observational study that evaluated the effect of cardiovascular drugs on the long-term survival of adults with one or more episodes of acute bronchitis. Cardioselective ß-blockers, but also calcium channel blockers, and statins may reduce all-cause mortality, an effect not seen with ACE-inhibitors or angiotensin receptor blockers. Apart from the infectious component of acute bronchitis, patients with such episodes can at least partly considered to have a chronic disease with a tendency to obstructive pulmonary disease and substantial cardiovascular comorbidities. Patients (22.7%) with cardiovascular comorbidities had significant higher mortality rates than those without (51.7% vs 12.0%, p<0.001). Interestingly, however, cardioselective ß-blockers, calcium channel blockers, and also statins reduced all-cause mortality to a similar extend in those with as without cardiovascular disease. This means that these drugs are also effective in patients with episodes of acute bronchitis who have other indication for these cardiovascular drugs, i.e. hypertension, hypercholesterolemia, and some arrhythmias.

Importantly, the indications to prescribe ACE-inhibitors/ARBs and calcium channel blockers overlap largely with the indications for prescribing ß-blockers. The indications to prescribe statins, however, differ from that of ß-blockers, and as such statins are not an alternative to ß-blockers. ß-blockers seem to perform better in preventing deterioration of cardiovascular diseases than ACE-inhibitors/ARBs. Our study results are in line with other observational studies performed in patients with chronic obstructive pulmonary disease (COPD), with consistent mortality reducing effects with cardioselective ß-blockers [14], [15], [22], but with neutral effects for calcium channel blockers [14], [23], and angiotensin-converting enzyme inhibitors/angiotensin receptor blockers [14], [16], [17]. A recent meta-analysis including nine observational studies evaluating the effect of ß-blockers in patients with COPD presented a pooled relative risk of mortality secondary to ß-blocker use of 0.69 (95% CI 0.62–0.78) [22]. Cardioselective ß-blockers seem to reduce mortality more than non-selective ß-blockers in patients with respiratory disease, although cardioselectivity decreases with higher dosing. In our study, the large majority used metoprolol at a moderate dose of 50–100 mg/day, a dosage compatible with cardioselectivity.

The beneficial effects of statins in our study could not only be related to cardiovascular effects, but at least also partly be explained by additional (pulmonary) anti-inflammatory effects [24].

### Limitations

In our cohort, some of the patients may have been misclassified with acute bronchitis, also because it is a diagnosis based on clinical grounds. Acute complaints of breathlessness and cough could have been caused by exacerbation of asthma or COPD. But also concealed ischemic heart disease with dyspnea as a symptom of angina pectoris, or exacerbations of heart failure in its early phases could also at least partly be the cause of such complaints [25]. This is important, because in these cardiovascular diseases the mortality-reducing effects of cardiovascular drugs are well established [26]. It is possible that we could not completely correct for confounding by indication in that more severely ill patients were more likely to receive cardiovascular drugs. Importantly, however, this confounding by indication would lead to underestimation of a beneficial treatment effect [27]. Although residual confounding by unobserved covariates cannot be completely ruled out, the hazard ratios of ß-blocker use in our study changed in the anticipated direction in the Cox analysis when different confounders were included one at a time. This suggests that potential unobserved confounders should be stronger than the observed ones that were put in our model to change the size of the effect, and it seems unlikely that such residual confounding exists [28]. We did not correct for smoking status, cholesterol level, or forced expiratory volume in 1 second (FEV1). We did, however, correct for statin use and assessed those with COPD in a subgroup analysis. In a previous observational study of ours smoking status was equally distributed between those on ß-blocker and COPD patients not on ß-blocker, and the adjusted hazard ratio for all cause mortality did not change when smoking was added as a potential confounder [14]. Therefore, these potential (residual) confounders seem not to have played an important role in our study. Confounding by contraindication could threaten the validity of ß-blockers in patients with COPD, because clinicians could knowingly withhold them in patients with more severe COPD due to concerns that the drug may worsen the patient's condition. Reserving prescription of ß-blocker to those with a less severe form of COPD and who may have a lower risk of mortality, could have resulted in confounding by contra-indication and therefore overestimation of the effect of ß-blockers. Importantly, however, confounding by contraindication with ß-blockers seems not to play a serious role in this study of patients with acute bronchitis.

We stopped our follow-up period on 01-01-2006 because we experienced difficulties with data extraction after the participating general practitioners changed their computer systems. Thus, observational studies on ß-blockers in COPD published after this date could not have influenced our data.

### Strengths

We included a large representative sample of adults with one or more episodes of acute bronchitis and had a long follow-up period. In our non-randomized study, we were able to correct for many potential confounders, and adjusted HRs were similar in important subgroups. Our cohort is a good representation of adults with episodes of acute bronchitis, including those who were (co-)treated by hospital specialists.

### Conclusions

The results of our study show that cardiovascular comorbities are common in adults who experience episodes of acute bronchitis. These persons should at least partly be considered to have a chronic (progressive) disease with a substantial risk to develop COPD and a rather high 5-years mortality risk. Cardioselective ß-blockers, but also calcium channel blockers, and statins may reduce all-cause mortality in these patients. Time has come to evaluate cardiovascular drugs in a broad range of patients with pulmonary complaints in a randomized, controlled trial. In the mean time, clinicians should actively investigate adult patients with pulmonary complaints for (early stages of) cardiovascular disease, and should realize that having pulmonary complaints not necessarily implies that a patient has a pulmonary disease. There are multiple common risk factors for lung and cardiovascular disease. Tobacco smoking, including second hand smoke, air pollution, and (desert or industrial) dust have clearly shown to have a negative effect on both the lungs and vascular endothelium, including the coronary system [29]–[32]. Desert dust, however, could not have played a role in our study because this type of dust is very unlike in the Netherlands. On the other hand, some epidemiological studies suggest that lower respiratory tract viral and bacterial infections can exhibit a (delayed or indirect) negative effect on the heart and vessels [33], [34].

Anyhow, time has come to consider any adult with pulmonary complaints to have (concealed or early stages of) cardiovascular disease, and one should without cardiovascular drugs, including cardioselective patients ß-blockers in these patients.
